# 

**Published:** 1926

**Authors:** 


### The Medical Library of the University of Bristol,

WITH WHICH IS INCORPORATED

### The Library of the Bristol Medico-Chirurgical Society.

---

*The following donations have been received since the publication of the List in June,* 1926.

*October*, 1926.

| | | |
|---|---|---|
| W. P. Eagleton, M.D. (1) | .. .. .. | 1 volume. |
| Professor E. Fawcett (2) | .. .. .. | 1 ,, |
| Professor E. W. Hey Groves (3) | .. .. | 4 volumes. |
| The Medical Officer of Health of Bristol (4) | .. | 1 volume. |
| The Registrar (5) | .. .. .. .. | 3 volumes. |
| Sir Humphry Rolleston (6) | .. .. .. | 1 volume. |
| The United Fruit Company (7) | .. .. | 2 volumes. |

Unbound periodicals have been received from Professor Hey Groves.

### THE ONE HUNDRED AND SEVENTEENTH LIST OF BOOKS.

The figures in round brackets refer to the figures after the names of the donors. The books to which no such figures are attached have either been bought from the Library Fund or received through the *Journal*.

| | | | |
|---|---|---|---|
| **Bassi, A.** .. .. .. .. | *Opere* .. .. .. .. .. .. .. .. .. .. .. .. .. | | 1925 |
| **Bland-Sutton, Sir J., and Giles, A. E.** | *Diseases of Women* | .. 8th Ed. | 1926 |
| **Browne, F. J.** .. .. .. | *Advice to Expectant Mother on the Care of her Health* .. .. .. .. .. .. .. .. .. .. .. .. .. | | 1926 |
| **Cathcart, G. C.** .. .. | *Tod's Diseases of the Ear* .. .. .. | .. 2nd Ed. | 1926 |
| **Cochrane, W. A.** .. | *Orthopædic Surgery* .. .. .. .. .. .. .. .. .. .. .. | | 1926 |
| **Cope, Z.** .. .. .. | *Treatment of the Acute Abdomen* .. .. .. .. .. | | 1926 |
| **Eagleton, W. P.** .. | *Cavernous Sinus Thrombophlebitis* .. .. .. .. | (1) | 1926 |
| **Fairbairn, J. S.** .. .. | *Obstetrics* .. .. .. .. .. .. .. .. .. .. .. .. .. .. | | 1926 |
| **Gibson, A. G.** .. .. | *The Heart* .. .. .. .. .. .. .. .. .. .. .. .. .. | | 1926 |
| **Graham, G.** .. .. | *Pathology and Treatment of Diabetes Mellitus* | 2nd Ed. | 1926 |

### LIBRARY

**Howard, R., and Perry, A.** *The House-Surgeon's Vade-Mecum* 2nd Ed. 1926
**Ibbotson, W.** .. .. *Surgical Operations* .. .. .. .. .. .. 2nd Ed. 1926
**Jordan, A. C.** .. .. *Chronic Intestinal Stasis, a Radiological Study* 2nd Ed. 1926
**Knaggs, R. L.** .. .. *Inflammatory and Toxic Diseases of Bone* .. .. 1926
**de Lint, J. G.** .. .. *Atlas of the History of Medicine. I. Anatomy* .. 1926
**MacKenzie, Sir James** *Principles of Diagnosis and Treatment in Heart Affections* .. .. .. .. .. .. .. 3rd Ed. 1926
**Mandl, F.** .. .. .. *Chirurgie der sportunfaelle* .. .. .. .. .. (3) 1925
**Pannett, C. A.** .. .. *Surgery of Gastro-duodenal Ulceration* .. .. .. 1926
**Pinch, A. E. H.** .. *Manual of Technique in Radium Therapy* .. (2) 1926
**Porritt, N.** .. .. .. *The Abdomen in Labour* .. .. .. .. .. .. .. 1926
**Riddell, J. R.** .. .. *Medical Electricity and Radiology* .. .. .. .. .. 1926
**Rolleston, Sir Humphry** *Jaundice, Diseases of Liver and Biliary Tract* (6) 1926
**Scott, R. J. E.** .. .. *Gould's Medical Dictionary* .. .. .. 8th Ed. 1926
**Snowman, J.** .. .. *Manual of Emergencies* .. .. .. 2nd Ed. (3) 1926
**Thomson, A., and Miles, A.** *Manual of Surgery.* 2 Vols. .. 2nd Ed. (3) 1926
**Thomson, F. G., and Gordon, R. G.** *Chronic Rheumatic Diseases* .. .. 1926
**Thomson, Sir St. Clair** *Diseases of the Throat* .. .. .. .. .. 3rd Ed. 1926
**Tomson, W. B.** .. .. *Notes and Suggestions for the Finding of Employment for the Tuberculous* .. .. .. .. .. .. 1926
**Vallery-Radot, P.** .. *Oeuvres de Pasteur.* Vol IV. .. .. .. .. .. 1926
**Watson, J. K.** .. .. *A Handbook for Nurses* .. .. .. .. .. 7th Ed. 1926
**Williams, L.** .. .. *Obesity* .. .. .. .. .. .. .. .. .. .. .. .. .. 1926

### TRANSACTIONS, REPORTS, JOURNALS, ETC.

Annual Report of the Medical Officer of Health, Bristol, 1925 .. .. (4) 1926
British Journal of Surgery, Vol. XIV., No. 53 (July) .. .. .. .. .. .. 1926
Cancer Review, Vol. I., No. 1 .. .. .. .. .. .. .. .. .. .. .. .. .. 1926
Collected Papers of the Mayo Clinic, Vol. XVII., 1925.. .. .. .. .. .. 1926
Dental Board of the United Kingdom: General Anæsthesia .. .. .. 1926
Guy's Hospital Reports, Vol. LXXVI., No. 3 (July) .. .. .. .. .. .. 1926
Minutes of General Medical Council, Vols. LIII. [1916], LIV. [1917], LV. [1918] (5)
Progressive Medicine (March) and (June) .. .. .. .. .. .. .. .. .. 1926
St. Bartholomew's Hospital Reports, Vol. LIX. .. .. .. .. .. .. .. 1926
Surgical Clinics of North America (June) .. .. .. .. .. .. .. .. .. 1926
United Fruit Company, Medical Department: Thirteenth and Fourteenth Annual Reports, 1924 and 1925 .. .. .. .. .. .. .. .. .. .. .. (7) —

### Local Medical Notes.

EXAMINATION RESULTS.

**University of Bristol.**—Students of the University have recently passed the following examinations :—

Bristol, L.D.S., R.C.S.—*First Professional—Part I. :* R. A. Phillips ; *Part II. :* R. A. Phillips, R. G. Boodle, C. Haskins, W. Woolley ; *Part III. :* A. F. N. Shapland. *Final—Part I. :* J. F. V. Sellin ; *Part II. :* A. W. M. Anderson, A. G. Batten, V. M. S. Jones, R. Y. Simpson, H. B. Smith.

L.S.A.—*Primary Examination (in Anatomy) :* G. Smith. *(In Physiology) :* L. P. Gregory, G. Smith.

F.R.C.S.—*Primary :* R. A. S. Cory, A. C. Fisher, K. H. Pridie. *Final :* F. Langford.

M.R.C.S., L.R.C.P.—G. L. Feneley.

University of London, M.B., B.S.—*Second Examination—Part II. :* P. Dalzell, L. E. Vine, K. H. Pridie. *Third Examination :* W. H. Royal.

